# ACO-Enhanced DCNN-BiLSTM framework for intrusion detection in smart Consumer Electronics network

**DOI:** 10.1371/journal.pone.0342949

**Published:** 2026-09-30

**Authors:** Jisi Chandroth, Gabriel Stoian, D.Jude Hemanth

**Affiliations:** 1 Department of AI and software, Gachon University, Seongnam, South Korea; 2 Department of Computer Science, University of Craiova, Craiova, Romania; 3 Department of ECE, Karunya Institute of Technology and Sciences, Coimbatore, India; Maulana Abul Kalam Azad University of Technology West Bengal, INDIA

## Abstract

Consumer Electronics (CE) devices, such as smartwatches, cameras, and smart home appliances, are becoming increasingly interconnected through Smart CE networks supported by Internet of Things (IoT) ecosystems and next-generation wireless networks. This ubiquitous connectivity enhances user convenience and enables intelligent services, but it also widens the attack surface by exposing resource-constrained devices to cyber threats. Although many CE devices constantly communicate with edge or cloud infrastructures, compromising a single vulnerable node can spread risk throughout the Smart CE network and jeopardize user privacy. Intrusion Detection Systems (IDS) are commonly used security systems for detecting threats and vulnerabilities in consumer devices. Although several IDS techniques have been developed in recent years, the Smart CE network environment still requires a real-time, highly accurate attack-detection solution to address its ever-changing, large-scale security concerns. In this paper, we propose a hybrid intrusion detection framework for securing smart CE network. The proposed model draws on the strengths and capabilities of multiple deep learning algorithms. Specifically, the proposed model combines a Deep Convolutional Neural Network (DCNN) and a Bidirectional Long Short-Term Memory (BiLSTM) network to accurately recognize threats. In addition, we use the Ant Colony Optimization (ACO) approach to extract informative and uncorrelated attributes. An attention layer is added to improve discriminative learning by emphasizing the most important representations. The proposed framework was evaluated using the UNSW-NB15 dataset. The experimental results show that the proposed framework is more accurate than the existing techniques.

## Introduction

IoT technology has grown significantly over the past few years. With the integration of IoT, conventional consumer electronics (CEs) have become more intelligent and automated. With the emergence of wearable and computing devices such as smartwatches and smartphones, CE devices can now be accessed remotely from anywhere and at any time [1]. The global consumer electronics market is estimated at USD 856.24 billion in 2025 and is expected to expand from USD 905.90 billion in 2026 to around USD 1,474.14 billion by 2035 [2]. Wearables are also moving beyond the wrist, creating new opportunities in computing, entertainment, and health. This segment is projected to grow by 32% to USD 56.6 billion by 2030, driven by emerging form factors such as smart rings, eyewear, and smart apparel. These devices can enable immersive entertainment and productivity, and ultimately reduce reliance on smartphones, laptops, and even televisions [3]. Communication protocols enable IoT connectivity among billions of CE devices and applications. These protocols also support integration with other domains, such as industry, healthcare, and transportation. The intelligent and autonomous capabilities of CE devices improve efficiency and enhance users’ quality of life. However, despite these advantages, smart CE networks face significant security and privacy risks because Internet-based connectivity exposes networked objects to malicious activities [4,5].

Consumer electronics devices, including wearables, home automation, and personal health gadgets, pose various kinds of security and privacy issues. For example, smartwatches and fitness trackers constantly capture sensitive physiological data (e.g., heart rate, sleep habits, and location), and a breach can reveal a user’s health profile and daily routines [6]. In home automation systems, compromised smart locks, lighting, or thermostats can enable attackers to control household functions, turn off safety settings, or determine whether a home is occupied [7]. Personal health equipment, such as smart glucose monitors or connected blood pressure meters, can be targeted to steal medical readings or interrupt regular operation, thereby compromising user well-being [8]. Voice assistants and connected hubs can also be used to eavesdrop, perform unwanted actions, or access linked accounts.

Intrusion Detection Systems are among the most promising security solutions for safeguarding consumer electronics devices and applications. An IDS can detect intrusive or malicious traffic within smart CE networks. However, many CE devices have limited storage and computational resources, which constrains the deployment of heavyweight security mechanisms. Therefore, dedicated and lightweight security measures are required to protect consumer applications against diverse attacks.

Traditionally, network operators relied on port-based or payload-based classification to detect intrusions. However, these old procedures have several disadvantages. Port-based classification labels traffic with well-known port numbers, but this assumption fails in heterogeneous smart CE networks where many apps employ dynamic or non-standard ports. Payload-based classification, on the other hand, relies on packet inspection and thus cannot successfully detect encrypted data. Since most smart CE network traffic are now encrypted, payload-based approaches become inefficient and often fail to identify such traffic correctly [9].

In recent years, Machine Learning (ML) and Deep Learning (DL) approaches have been widely used in network security. However, the distributed architecture of smart CE networks often produces noisier, more diverse data, rendering typical ML algorithms insufficient for accurate attack detection. Although machine learning approaches can extract latent patterns from traffic characteristics for categorization, acquiring high-quality traffic features takes time and effort. Due to domain-specific characteristics, limited resources, stringent network requirements, and diverse protocol stacks, implementing IDS solutions using traditional ML is difficult in smart CE networks [10]. Deep learning algorithms have also gained popularity for their ability to extract features and learn complex nonlinear representations automatically. However, DL models have poor generalization and limited robustness, especially in highly dynamic and diverse smart CE network scenarios when available traffic data is scarce or insufficiently representative [11]. The limitations discussed above motivate us to develop a secure IDS solution capable of accurately detecting threats and attacks in smart CE networks.

This work aims to enhance intrusion detection accuracy by accounting for the dynamic, heterogeneous, and resource-constrained characteristics of smart CE networks. The main objective is to create a hybrid IDS architecture that can identify threats with minimal detection latency. To do this, we create a hybrid deep-learning architecture that includes a DCNN with a BiLSTM, enabling the model to learn complementary spatial and temporal features of smart CE network traffic flows. In addition, an attention strategy is used to highlight the most discriminative representations. In contrast, Ant Colony Optimization (ACO) is used to extract low-correlation features, minimizing redundancy and increasing efficiency.

The main contributions of the work are stated below:

- The paper proposes a hybrid deep-learning architecture that combines a DCNN with a BiLSTM, enabling the model to learn both spatial and temporal features of CE-IoT traffic flows and to detect intrusions with minimal latency.
- To address the high-dimensional and heterogeneous data problem, an Ant Colony Optimization (ACO) algorithm is introduced to select less-correlated features.
- A temporal attention layer is employed to highlight the most discriminative representations and improve class-level separation.
- Compared with existing methods, the proposed scheme more effectively detects network intrusions and achieves improved classification performance.

## Literature review

The literature review section contains several IDS-related past research works using ML and DL algorithms. Recently, federated learning and explainable AI have gained a lot of attention. Other works include ensemble learning frameworks, and feature selection techniques. A comparative summary of the reviewed studies is provided in Table 1.

**Table 1:** Comparison of existing intrusion detection models and limitations.

Ref.	Model	Limitations
[1]	One-Dimensional Convolutional Neural Network (1D-CNN)	Potential activation leakage and high communication overhead arising from iterative client–server interactions.
[4]	Hybrid CNN–LSTM	Limited cloud coordination support and high computational complexity
[12]	Multi-scale bidirectional GRU	High computational latency and resource overhead from iterative preprocessing and complex bidirectional recurrent layers.
[13]	Semi-supervised GCN–GAT-based FIR-GNN	High graph-memory overhead, sensitivity to time-window settings, and poor performance on minority attack classes.
[14]	Kullback Gaussian Deep Belief Network	Manual hyperparameter tuning, sensitivity to class imbalance, and lack of validation in real-world operational network environments.
[15]	RF, ANN, LSTM, and GRU classifiers.	Performance degradation on specific attacks, sensitivity to class imbalance, and dependence on limited satellite traffic datasets.
[16]	CNN-LSTM	High computational overhead, privacy concerns, vulnerability to adversarial and zero-day attacks, and limited cross-domain generalization.
[17]	Two stage BiLSTM	Limited cross-dataset validation, inability to adapt to concept drift and zero-day attacks.
[18]	Protocol-Aware Auxiliary Classifier GAN + XG Boost	Limited validation on benchmark datasets
[5]	Attention-Based Deep Reinforcement Learning	Blockchain scalability constraints, limited capability to capture complex feature interactions
[19]	Quantum Federated Learning with Parameterized Quantum Circuits and Quantum Neural Network	Fragile qubits, noisy intermediate-scale quantum hardware limitations, quantum data encoding overhead, and dependence on a hybrid quantum–classical architecture.
[20]	Personalized Federated Hypernetworks	Dependence on reliable gradient exchange, risk of gradient-based information leakage, complex multi-level optimization, and reduced adaptability to rapidly evolving attack patterns.
[21]	Auto-Encoder Neural Support Vector Machine with Multi-View Federated Learning	High model complexity, sensitivity to class imbalance, lower performance than centralized learning, and slightly inferior classification performance compared with specialized convolutional neural network models.
[22]	Federated Meta-Learning with Digital Twins and Soft Actor-Critic Reinforcement Learning	High computational complexity, continuous client monitoring and clustering overhead, and vulnerability to gradient-based privacy attacks such as model inversion and membership inference.
[23]	Contrastive Learning + Isolation Forest + Gaussian Mixture Model	Limited stealth attack detection, hyperparameter sensitivity, and reduced performance under dynamic client participation.
[24]	Two-Phase Feature Selection Framework	Limited dataset validation, dependence on Shapley Additive Explanations, and no deep feature extraction.
[25]	ACO-Based Feature Selection with Support Vector Machine	Sensitive to parameter tuning, trade-off between accuracy and feature reduction, and slow Support Vector Machine training on large datasets.
[26]	Combinatorial Multi-Classification Neural Network	High computational cost, long training time, sensitivity to feature loss, and limited effectiveness of conventional multi-class neural network training.
[27]	ACO-Based CNN with Dynamic Voltage and Frequency Scaling	Binary classification only, high computational complexity, overfitting risk, and sensitivity to parameter tuning.
[28]	Support Vector Regression with Hybrid ACO-Based Ensemble Feature Selection	Parameter sensitivity, no convergence guarantee, limited nonlinear feature modeling, and inconsistent statistical feature rankings.
[29]	Genetic Algorithm-Optimized XGBoost Ensemble Classifier	Limited to known attacks, lacks real-time validation, and depends on randomized metaheuristic optimization.

### ML, DL and hybrid IDS approaches

Several IDS approaches have been developed using ML and DL algorithms. In [1], the authors proposed a Software Defined Network (SDN)-orchestrated split-learning anomaly-detection framework for CE networks in smart environments, where a 1D-CNN is deployed in the control plane. However, it achieves strong accuracy, precision, recall, and F1-score on CICIDS-2018; privacy leakage is a noted limitation. In [4], a blended CNN-LSTM framework is introduced for the consumable edge-centric Internet of Medical Things (IoMT) industry to detect new intrusions, reporting 98.53% accuracy. However, the evaluation is limited to a single dataset and does not analyze detection time. In [12], an IDS model based on a Multi-scale Deep Bidirectional Gated Recurrent Neural Network (MDBGRNN) is proposed for IoT security, with reported limitations in scalability and sensitivity to data quality. On the other hand, work in [13] presents a Feature-Importance-Refined Graph Neural Network (FIR-GNN) for Cyber-Physical Systems (CPS) smart homes, using a directed graph with edge-feature self-attention and semi-supervised learning. At the same time, SHapley Additive exPlanations (SHAP)-based feature subset selection reduces computational cost and improves accuracy, precision, recall, and F1-score.

The work in [14] proposes an intrusion detection approach based on an improved Deep Belief Network (DBN). First, the probabilistic mass function (PMF) and Min–Max methods are introduced to simplify data preprocessing. Then, a combined sparsity penalty term is incorporated into the likelihood function during the unsupervised learning phase. By imposing sparse constraints, the model encourages sparse activation of hidden-layer neurons, thereby reducing feature homogeneity and mitigating network overfitting. Experimental results show that the proposed approach achieves satisfactory performance on large-scale network datasets. However, most DBN structural parameters must be determined through extensive experimentation, and uncertainty in parameter selection may affect detection accuracy. To provide a high level of security for both satellite and terrestrial networks [15], proposes four hybrid IDS models based on ML and DL techniques. A sequential forward selection (SFS) method with Random Forest (RF)-based feature selection is used to improve classification performance and reduce execution time. The selected features are then combined with different learning models, including RF, LSTM, Artificial Neural Network (ANN), and Gated Recurrent Unit (GRU). The reported results indicate that the proposed hybrid models are more efficient than prior work.

Several intrusion detection systems have been proposed for IoT environments using advanced deep learning and data augmentation techniques. In [16], a multimodal NIDS combines transfer learning, attention-based ResNet, and CNN-LSTM architectures to learn both semantic and visual representations of network traffic, achieving high detection performance. However, the framework introduces considerable computational overhead due to complex feature extraction and multimodal processing. TIDE-Net [17] employs a two-stage deep learning architecture for IoT intrusion detection, where traffic is first classified as benign or malicious and then categorized into specific attack types using BiLSTM-based models. Although the framework achieves approximately 97% end-to-end accuracy, error propagation between stages may affect overall reliability. To address class imbalance, the PA-ACGAN-XGBoost framework [18] generates protocol-aware synthetic traffic samples and trains an XGBoost classifier for improved attack detection. While effective for minority-class recognition, the computational complexity of generative models remains a challenge for resource-constrained IoT deployments. These studies demonstrate the effectiveness of deep learning for IoT intrusion detection while highlighting the need for lightweight and computationally efficient solutions.

### Explainable AI-based IDS approaches

Nowadays, explainable AI (XAI) has been in the spotlight for its ability to enhance transparency and trust in AI-driven security systems. In [5], the authors devised a security framework for innovative consumer applications by integrating blockchain-driven Authorization and Key Agreement (AKA) mechanisms with an XAI-enabled IDS. Initially, entity registration and authentication are performed within the network, and a Proof-of-Authority (PoA) blockchain consensus mechanism is used to validate and verify authenticated data. For attack detection, the framework proposes an XAI-based IDS that leverages deep reinforcement learning (DRL), incorporating an attention mechanism with a softmax activation function to focus on relevant traffic features and accurately identify malicious activities. To address the black-box nature of AI-based IDS solutions, the study uses the Python-based SHAP framework to explain and interpret the most influential features driving IDS decisions.

### Federated learning-based IDS approaches

Recent studies increasingly adopt Federated Learning (FL) for intrusion detection to address privacy, scalability, and heterogeneity in IoT and consumer networks. For example, QFL-IDS [19] combines FL with quantum computing to collaboratively train a global IDS across distributed consumer devices while preserving local data privacy and improving training efficiency. Conventional FL has key limitations, including the need to enforce a shared model architecture across Non-IID heterogeneous data and the high communication overhead of weight-based aggregation. Security protections such as homomorphic encryption and differential privacy further increase computational cost and may still expose data-distribution patterns if compromised. To address these issues, PerFedHypID [20] uses a personalized federated hypernetwork that aggregates lightweight embedding vectors rather than full model weights, improving client-specific adaptation at lower communication cost. However, extreme and rapid concept drift remains challenging. Moving beyond single-view learning [21], introduces a multi-view fusion FL framework using an AE-NSVM model. In which autoencoders learn compact representations from multiple feature views and an SVM performs multi-class attack classification, improving robustness across diverse clients and motivating hierarchical client–edge–cloud deployments. The work [22] targets 6G-enabled consumer electronics networks, where intrusion detection is difficult due to highly dynamic traffic and severe class imbalance. It proposes a federated meta-learning IDS integrated with digital twins in a Meta-Verse environment to support distributed and adaptive learning. This design enables scalable, controlled experimentation and improves intrusion-detection performance under highly imbalanced, evolving attack conditions. Moreover, the work done in [23] introduces a server-side framework that defends federated learning in open-world environments by using a multi-stage process of contrastive representation learning and pseudo-labeling to filter malicious updates without requiring trusted data. The system stabilizes detection through an adaptive thresholding mechanism, relying on reputation scores and consecutive-confirmation rules to ensure reliability. However, the framework’s effectiveness is limited if attackers perform highly subtle, stealthy poisoning that blends into natural data variability.

### Feature selection-based IDS approaches

Feature selection (FS) is very important for both ML and DL algorithms [24]. Although DL models can learn features automatically, applying additional feature selection can still improve accuracy because it removes irrelevant and noisy attributes, reduces redundancy among correlated features, and helps the model focus on the most discriminative patterns. Genetic and swarm-based metaheuristics (e.g., ACO, PSO, GA) are effective for feature selection because they can efficiently search very large feature spaces and identify near-optimal subsets without exhaustive evaluation [25,26]. They also capture complex, non-linear feature interactions while balancing high accuracy with fewer selected features. ACO-based intrusion detection has received less attention than other popular metaheuristics, such as swarm intelligence approaches [27,28]. One issue is that ACO can be computationally expensive to test and may not scale effectively to very high-dimensional traffic data unless the algorithm is carefully designed. Furthermore, current trends in IDS research have shifted toward XAI and FL, with less emphasis on metaheuristic feature selection. Nonetheless, ACO remains a promising solution, especially for lightweight IDS solutions that require a compact, high-impact feature set [29].

The review of the literature shows that the current research focuses on deep learning architecture, explainable AI, federated learning or feature selection separately. While CNN-LSTM and similar deep learning models have shown high performance in intrusion detection, most existing work either considers the full feature space or applies traditional feature reduction methods. Similarly, attention mechanisms, explainable AI and federated learning have been explored individually to enhance interpretability, privacy and scalability. However, the integration of ACO based feature optimization with attention enhanced spatiotemporal deep learning models has received less attention. Thus, there is a research gap in developing a unified IDS framework that simultaneously reduces feature redundancy, captures spatial and temporal traffic characteristics, and improves attack detection. To bridge this gap, this work proposes an ACO enhanced DCNN-BiLSTM framework with a temporal attention mechanism for intrusion detection.

## Proposed work

This section discusses the proposed hybrid IDS framework that integrates two deep learning architectures to detect attacks in the Smart CE environment effectively. In addition, the framework incorporates ACO for feature selection. A complete overview of the proposed model is shown in Fig 1. This section covers the ACO-based feature selection process, model training, and performance evaluation.

**Fig 1:**
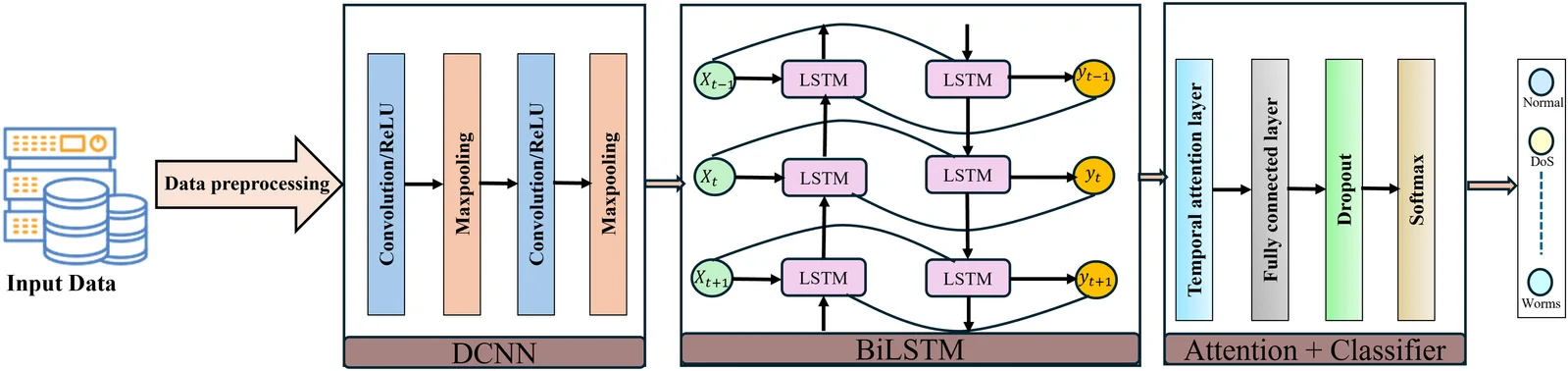
Overall framework of the proposed IDS Model.

### Data preprocessing

The raw dataset contains missing values, duplicates, and noise, therefore, preprocessing the data before model training is essential. In this work, we perform data cleaning and apply label encoding and Min–Max normalization to make the data suitable for model training. Missing values are addressed by deleting records with missing labels. Duplicate records are eliminated to prevent bias in learning. In addition, noise and inconsistent values are removed to improve data quality. Following that, label encoding converts categorical attributes to numerical values by assigning a unique integer to each category, enabling the model to handle non-numeric fields. After encoding, the data is scaled using Min-Max normalization to ensure that all numeric features are within a consistent range. This avoids features with higher values from dominating the learning process, allowing the model to converge more consistently during training.

### ACO feature selection

Dimensionality reduction is significant in deep learning models because it reduces the number of redundant attributes, lowering time complexity and increasing model accuracy by selecting the most important features. An overfitting problem arises during training when a large number of features are discovered. In this study, the Ant Colony Optimization Algorithm (ACO) is employed to select uncorrelated attributes. The ACO method is highly robust and performs well on complex optimization problems, and it is cutting-edge for dimension-reduction optimization problems [30]. This optimal feature subset search is an ant path through graph where the minimum number of the visited nodes is suitable with the traversal stopping criterion.

Let the preprocessed intrusion detection dataset be denoted as:


$$\begin{array}{c} 𝒟={\left\{({𝐱}^{\left(i\right)},y^{\left(i\right)})\right\}}_{i=1}^{N}, \end{array}$$ (1)

where *N* is the number of network traffic instances, ${𝐱}^{\left(i\right)}\in {\mathbb{R}}^{F}$ is the original feature vector with *F* features, and *y*^(*i*)^ is the corresponding class label. Specifically,


$$\begin{array}{c} {𝐱}^{\left(i\right)}={\left[x_{1}^{\left(i\right)},x_{2}^{\left(i\right)},...,x_{F}^{\left(i\right)}\right]}^{⊤}. \end{array}$$ (2)

The objective of ACO is to select a compact feature subset *d*<<*F* while preserving high intrusion detection performance. ACO formulates feature selection as a path-construction problem, each ant incrementally builds a candidate subset by traversing a graph whose nodes represent features. In this setting, searching for the optimal subset is equivalent to finding an ant path that yields the best fitness (macro-F1) with as few visited nodes as possible. The traversal can be terminated once the subset size reaches *d* or when no further improvement is observed, ensuring a compact yet discriminative feature set.

Each candidate feature subset is encoded by a binary selection vector


$$\begin{array}{c} 𝐱=\left[x_{1},x_{2},...,x_{F}\right],\;x_{j}\in \{0,1\},\;j=1,...,F, \end{array}$$ (3)

where $x_{j}=1$ indicates that the *j*-th feature is selected and $x_{j}=0$ indicates it is excluded. The number of selected features is


$$\begin{array}{c} d=\sum_{j=1}^{F}x_{j},\;d\ll F, \end{array}$$ (4)

The ACO algorithm is based on the pheromone-based communication system used by ants. Ants use pheromone trails to signal and select features based on their intensity. Routes with high pheromone concentration become more efficient over time, guiding the ant colony toward optimal solutions.

For each feature *j*, ACO maintains a pheromone value $\tau_{j}(t)$ at iteration *t* and a heuristic desirability $\eta_{j}$. The probability of selecting feature *j* is defined as:


$$\begin{array}{c} p_{j}(t)=\frac{\tau_{j}(t{)}^{\alpha }\;\eta_{j}^{\beta }}{\sum_{k=1}^{F}\tau_{k}(t{)}^{\alpha }\;\eta_{k}^{\beta }}, \end{array}$$ (5)

where α and β control the relative influence of pheromone and heuristic information, respectively. Each ant constructs a solution vector ${𝐱}^{\left(a\right)}$ by sampling features according to $\{p_{j}(t){\}}_{j=1}^{F}$ such that the subset size satisfies $\sum_{j=1}^{F}x_{j}^{\left(a\right)}=d$.

Each feature subset generated by an ant is evaluated by training a lightweight classifier and computing a balanced performance metric. The fitness function is defined as:


$$\begin{array}{c} Fit(𝐱)={F1}_{macro}(𝐱)-\lambda \frac{d}{F}, \end{array}$$ (6)

where ${F1}_{macro}$ is the macro-averaged F1-score and λ penalizes overly large feature subsets.

Pheromone values are updated using evaporation and reinforcement:


$$\begin{array}{c} \tau_{j}(t+1)=(1-\rho )\tau_{j}(t)+\Delta \tau_{j}(t), \end{array}$$ (7)

where ρ∈(0,1) is the evaporation rate and


$$\begin{array}{c} \Delta \tau_{j}(t)=\sum_{a=1}^{A}𝕀\;\left(x_{j}^{\left(a\right)}=1\right)\cdot Fit\;\left({𝐱}^{\left(a\right)}\right), \end{array}$$ (8)

with *A* being the number of ants and 𝕀(·) the indicator function.

After *T* iterations, the optimal feature subset is selected as


$$\begin{array}{c} {𝐱}^{*}=\text{arg}\max_{𝐱}Fit(𝐱), \end{array}$$ (9)

### DCNN-BiLSTM and Temporal Attention based IDS

After ACO, the selected feature subset is fed into the DCNN-BiLSTM module to extract discriminative local feature patterns. A Deep Convolutional Neural Network (DCNN) is first employed. The DCNN consists of two one-dimensional convolutional layers with a kernel size of 3 and ReLU activation. The first convolutional layer applies 64 filters to the input sequence, followed by a max pooling operation with a pool size of 2 to reduce dimensionality. The second convolutional layer applies 128 filters, followed by max pooling. The DCNN extracts hierarchical feature representations through successive convolution and pooling operations, producing intermediate feature maps defined as follows:


$$\begin{array}{c} U_{1}=ReLU\;\left({Conv1D}_{64}(X)\right) \end{array}$$ (10)

$$\begin{array}{c} P_{1}=MaxPool(U_{1}) \end{array}$$ (11)

$$\begin{array}{c} U_{2}=ReLU\;\left({Conv1D}_{128}(P_{1})\right) \end{array}$$ (12)

$$\begin{array}{c} P_{2}=MaxPool(U_{2}) \end{array}$$ (13)

where, *X* is the ACO feature matrix reshaped as a one-dimensional sequence, *U*_1_, and *U*_2_ are the output of the first and second convolutional layer, obtained by applying a one-dimensional convolution with learnable weights and bias followed by a ReLU activation function. *P*_1_ and *P*_2_ are the pooled feature representation after the first and second max pooling operation.

The extracted feature sequence is then processed by a Bidirectional Long Short-Term Memory (BiLSTM) network to model contextual dependencies in both forward and backward directions. The BiLSTM captures long-range correlations among traffic features that are not explicitly modeled by convolutional layers. The BiLSTM output is given by:


$$\begin{array}{c} 𝐇=BiLSTM({𝐏}_{2}), \end{array}$$ (14)

where


$$\begin{array}{c} 𝐇=\{{𝐡}_{t}{\}}_{t=1}^{T^{\prime }},\;{𝐡}_{t}\in {\mathbb{R}}^{256}. \end{array}$$ (15)

Each hidden state ${𝐡}_{t}$ is obtained by concatenating the forward and backward LSTM outputs, with 128 hidden units in each direction.

To enhance the discriminative capability of the model, a temporal attention mechanism is applied to the BiLSTM outputs. The attention score for each hidden state is computed as:


$$\begin{array}{c} e_{t}={𝐯}^{⊤}\tanh(𝐖{𝐡}_{t}+𝐛), \end{array}$$ (16)

where 𝐖 and 𝐯 are trainable weight parameters and 𝐛 is a bias vector.

The output of the attention layer is then passed to a fully connected classifier. A dense layer with 128 neurons and ReLU activation is applied:


$$\begin{array}{c} 𝐠=ReLU({𝐖}_{1}𝐳+{𝐛}_{1}), \end{array}$$ (17)

followed by a softmax output layer that produces the predicted class probabilities:


$$\begin{array}{c} \hat{𝐲}=softmax({𝐖}_{2}𝐠+{𝐛}_{2}), \end{array}$$ (18)

where $\hat{𝐲}\in {\mathbb{R}}^{C}$ denotes the probability distribution over *C* traffic classes. The network parameters are optimized during the training phase by minimizing the categorical cross-entropy loss using the Adam optimizer:


$$\begin{array}{c} ℒ=-\frac{1}{N}\sum_{i=1}^{N}\sum_{c=1}^{C}y_{c}^{\left(i\right)}\log{\hat{y}}_{c}^{\left(i\right)}, \end{array}$$ (19)

where $y_{c}^{\left(i\right)}$ is the ground-truth label of class *c* for the *i*-th instance.

### Performance evaluation

This section details the experimental setting, the dataset, and the evaluation metrics used. After that, we provide a complete analysis of the obtained results, followed by a comparison with existing research to demonstrate the effectiveness of the proposed method.

### System requirements

We implemented the proposed method in Python using TensorFlow, Keras, and Scikit-learn to develop and evaluate the model. All experiments were performed on a machine equipped with a 12th-generation Intel(R) Core(TM) i5-12600K processor (3.69 GHz) and 48 GB of RAM.

### Dataset description

We evaluated the proposed intrusion detection framework using the UNSW-NB15 dataset [31], which is one of the most widely adopted benchmark datasets for IDS evaluation. It was developed by the University of New South Wales (UNSW), Australia, the dataset contains approximately 2.5 million network traffic records collected in a realistic simulated environment comprising both normal and malicious activities. The dataset was selected because it provides a realistic representation of contemporary network traffic and cyberattack scenarios that are highly relevant to smart consumer electronics environments. It includes diverse attack categories such as denial-of-service (DoS), worms, and probing, which closely resemble threats that target smart consumer devices, including smart home appliances, wearable devices, surveillance systems, smart televisions, and IoT gateways. Furthermore, the dataset incorporates a comprehensive set of flow-based and packet-level features that capture communication patterns commonly observed in interconnected CE networks. Since modern consumer electronics increasingly rely on edge and cloud connectivity, the heterogeneous traffic characteristics and attack diversity of UNSW-NB15 make it an appropriate benchmark for evaluating intrusion detection systems in CE-IoT ecosystems. Notably, the dataset is highly imbalanced, and no class-balancing approaches were used in this study. For evaluation, the data were split into 70% for training, 15% for validation, and 15% for testing. Table 2 shows the Percentage-wise distribution of network traffic classes including normal and attack categories in the UNSW-NB15 dataset.

**Table 2:** Percentage-wise Distribution of Network Traffic Classes.

Attack Class	Instances (%)
Normal	44.94
Generic	22.92
Exploits	13.52
Fuzzers	7.36
DoS	4.966
Reconnaissance	4.246
Analysis	0.822
Backdoor	0.708
Shellcode	0.459
Worms	0.053

### Training details

For performance evaluation, the dataset was divided into 70% training, 15% validation, and 15% testing subsets. The training set was used to train the proposed model, while the validation set was utilized for hyperparameter tuning, model selection, and ACO-based feature subset evaluation. The test set was used to evaluate the final model’s performance. For feature selection, the ACO algorithm was configured with *A* = 10 ants and a maximum of *T* = 15 iterations. The pheromone evaporation rate was set to ρ=0.3, while the relative influence of pheromone trails and heuristic information was controlled using α=1 and β=2, respectively. The heuristic value was defined as the variance of each feature computed from the training data ($\eta_{j}=Var(x_{j})$). Candidate feature subsets were evaluated using a lightweight multinomial logistic regression classifier with a maximum of 200 iterations. The fitness function was defined as the macro-F1 score penalized by feature subset size using a penalty coefficient of (λ=0.001). The stopping criterion was a fixed maximum of 15 iterations. Furthermore, feature selection was performed using the training data and validated on the validation set, while the test set was excluded from the feature-selection process to prevent test-data leakage. To reduce overfitting, dropout was applied with a rate of 0.3. Model parameters were optimized using the Adam optimizer with a learning rate of 10^–3^, $\beta_{1}=0.9$, $\beta_{2}=0.999$, and $\epsilon =10^{-8}$. The model was trained by minimizing the categorical cross-entropy loss function for a maximum of 35 epochs, using a batch size of 64.

### Evaluation metrics

To assess the effectiveness of the proposed framework, we used standard machine-learning evaluation metrics, namely accuracy, precision, recall, and F1-score. For each class, correctly predicted samples are counted as True Positives (TP), while samples correctly identified as not belonging to that class are counted as True Negatives (TN). Misclassifications are captured by False Positives (FP), which represent samples incorrectly assigned to the class, and False Negatives (FN), which denote samples from the class that the model failed to detect. Table 3 summarizes the different evaluation metrics and their equations.

**Table 3:** Evaluation metrics.

Metric	Equation
Accuracy	$\frac{TP+TN}{TP+TN+FP+FN}$
Precision	$\frac{TP}{TP+FP}$
Recall	$\frac{TP}{TP+FN}$
F1-score	$\frac{2\cdot Precision\cdot Recall}{Precision+Recall}$

## Results

This subsection analyzed the outcome of the proposed framework. The Fig 2 depicts the behavior of the ACO feature-selection process by plotting the distribution of ant fitness values (Macro-F1) at each iteration with boxplots. Each box represents the performance of all ants during a given iteration, revealing both the quality of possible feature subsets and the overall search diversity. The results show substantial dispersion in the early stages, especially at iteration 2, indicating a broad exploration of the solution space and the presence of multiple low-performing subsets. From iterations 3–5 onward, the distributions become densely concentrated around a single Macro-F1 value, indicating that the colony rapidly reinforces strong feature combinations via pheromone updates and switches to exploitation, with limited variation across ants. The uniform medians and minimal dispersion in later iterations indicate stable convergence and diminishing performance benefits, showing that the ACO method identifies a consistent feature subset in a limited number of iterations.

**Fig 2:**
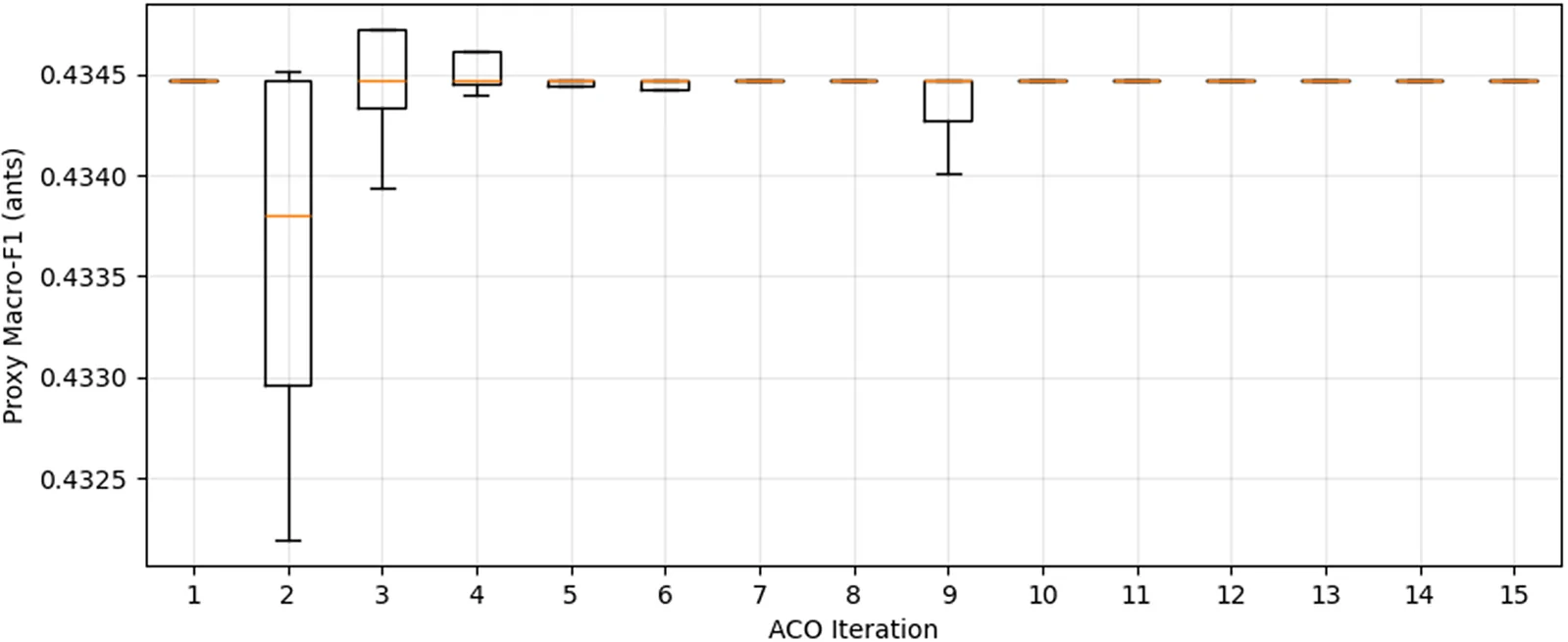
ACO search distribution across iterations.

Fig 3 shows the confusion matrix of the proposed method. The proposed method accurately classifies the dominant traffic classes, but its performance degrades on minority attack classes. Specifically, the “Normal” class is ideally classified, and the “Generic” class achieves near-perfect recognition with only a small number of misclassifications. “Fuzzers” also shows high detection capability, although a fraction of samples are confused with “DoS”, “Exploits”, and “Reconnaissance”. The most notable error pattern is the mutual confusion between “DoS” and “Exploits”, where a considerable number of “DoS” instances are predicted as “Exploits” and vice versa. In contrast, rare classes such as “Analysis”, “Backdoor”, “Shellcode”, and “Worms” exhibit low recall.

**Fig 3:**
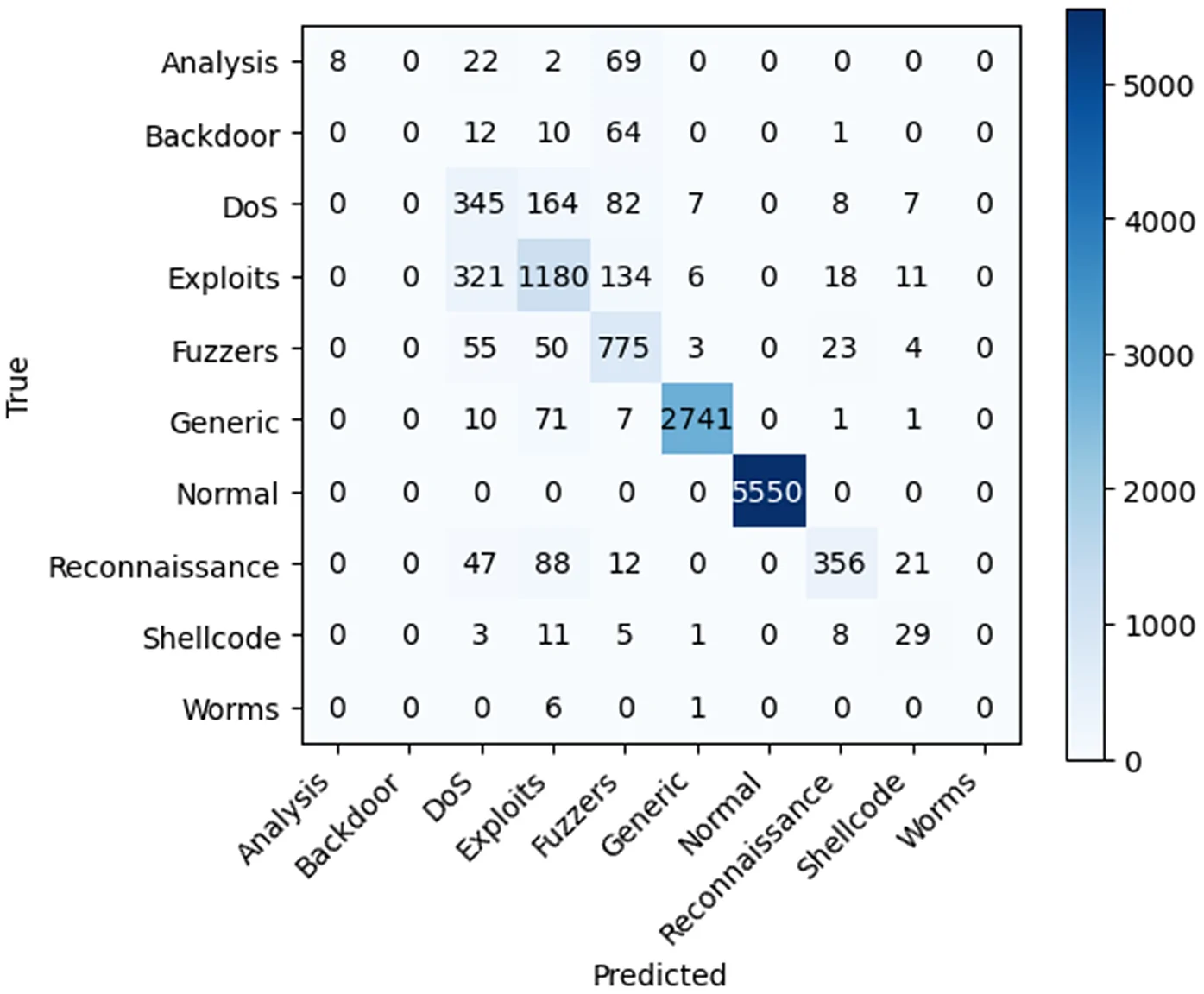
Confusion Matrix of all Attacks on UNSW-NB15 Dataset.

The obtained results are presented in Fig 4 and 5. The graphical representation of the results indicates a stable training and validation performance with the number of epochs is changed. Fig 6 illustrates the distribution of inference time per batch. The results show a narrow, unimodal distribution centered at 0.08–0.11 seconds, indicating stable, and predictable inference latency. The limited variance and absence of significant outliers demonstrate the suitability of the proposed model for real-time intrusion detection in smart CE network environments.

**Fig 4:**
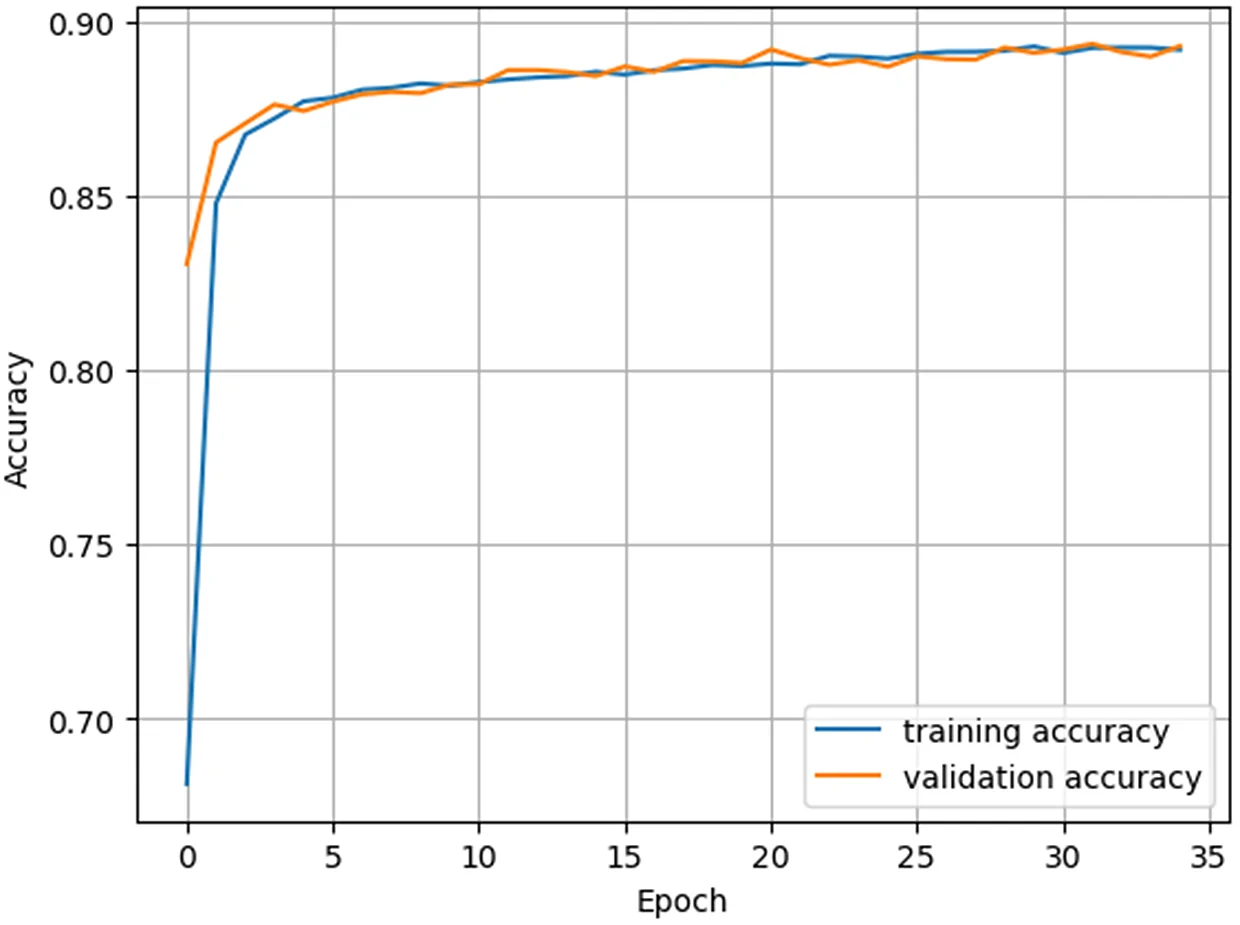
Accuracy of proposed method on UNSW-NB15 Dataset.

**Fig 5:**
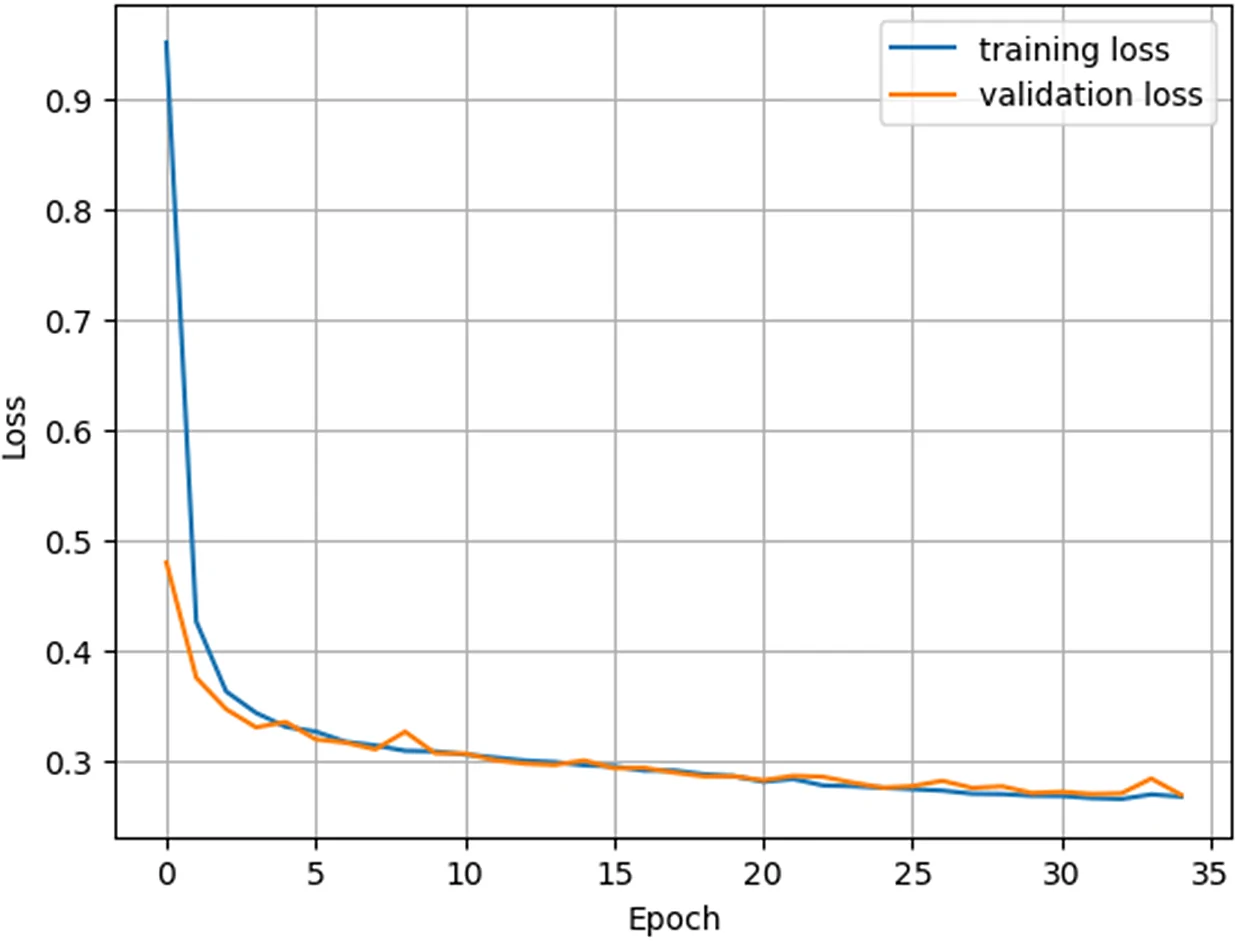
Loss of proposed method on UNSW-NB15 Dataset.

**Fig 6:**
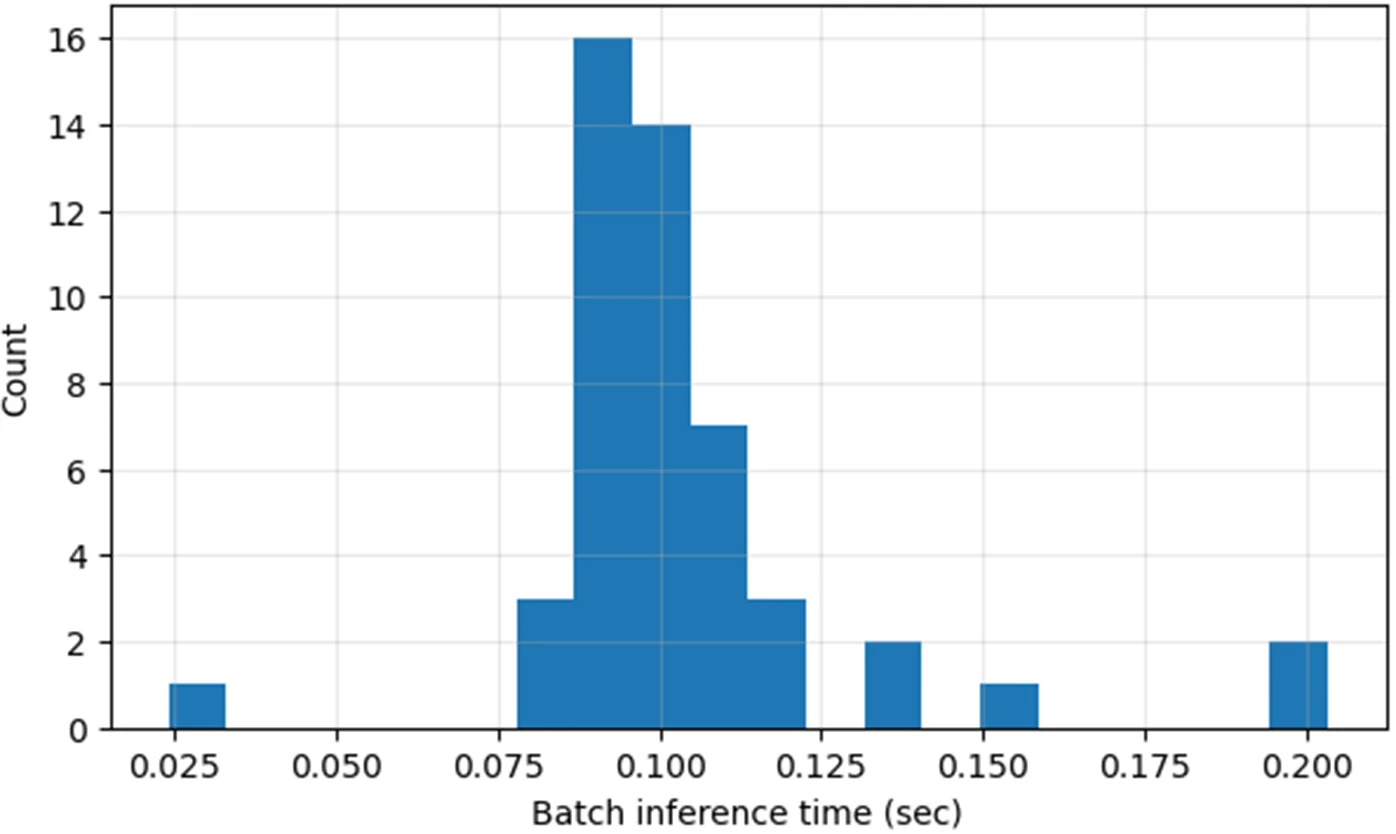
Distribution of inference time per batch of the proposed model.

Table 4 compares the performance of the proposed intrusion detection framework with several recent IDS methods reported in the literature, all evaluated on the UNSW-NB15 dataset. The comparison is conducted using four widely adopted metrics such as accuracy, precision, recall, and F1-score. Overall, the proposed method achieves 89.04% accuracy, 88.93% precision, 89.04% recall, and an F1-score of 88.72%, indicating a strong and well-balanced detection performance. In contrast, many existing approaches either yield lower overall results or show imbalance between precision and recall, which can lead to missed attacks.

**Table 4:** The Comparison on Different IDS Methods with Proposed Framework.

Ref	Year	Accuracy (%)	Precision	Recall	F1 score
[20]	2025	84.4	82.3	86.2	84.0
[21]	2025	–	79.9	85.4	82.6
[15]	2023	79	67	63	62
[14]	2020	86.49	76.32	51.05	53.92
[29]	2024	82.52	85.72	82.52	84.08
[24]	2025	88.83	–	–	–
[22]	2024	84	89.03	78.96	83
Proposed	2026	89.04	88.93	89.04	88.72

Table 5 presents an ablation study evaluating the contributions of the key components of the proposed system. Among the baseline models, the BiLSTM-only configuration achieves the lowest accuracy of 54.29%, indicating that temporal modeling alone is insufficient for effective intrusion detection. The DCNN-only model improves the accuracy to 84.72% by capturing discriminative spatial patterns from network traffic features. Combining DCNN and BiLSTM further increases the accuracy to 87.56%, demonstrating the benefit of jointly learning spatial and temporal representations.

**Table 5:** Ablation study of the proposed framework.

Model	ACO	DCNN	BiLSTM	Attention	Accuracy (%)	Training Time (s)
DCNN Only	No	Yes	No	No	84.72	397.5341
BiLSTM Only	No	No	Yes	No	54.29	597.4216
DCNN + BiLSTM	No	Yes	Yes	No	87.56	1245.2373
Without ACO	No	Yes	Yes	Yes	87.89	685.9395
Without DCNN	Yes	No	Yes	Yes	87.74	–
Without BiLSTM	Yes	Yes	No	Yes	88.87	–
Without Attention	Yes	Yes	Yes	No	87.44	252.5970
Full Model	Yes	Yes	Yes	Yes	89.04	3301.1489

The complete model, which integrates ACO-based feature selection, DCNN, BiLSTM, and the temporal attention mechanism, achieves the highest accuracy of 89.04%, demonstrating the effectiveness of the overall architecture. Removing the ACO module drops accuracy to 87.89%, which indicates that ACO-based feature optimization eliminates redundant features and improves model performance. The ACO-based feature selection module increases classification accuracy from 87.89% to 89.04%, indicating that improving the input feature subset enhances the model’s decision-making ability. Network traffic datasets sometimes include duplicate and correlated features, which can generate noise and raise computational complexity. ACO eliminates feature redundancy by selecting only the most useful features, enabling the deep learning model to focus on traffic characteristics most relevant to attack detection.

Similarly, removing the DCNN component reduces the accuracy to 87.74%, confirming the importance of spatial feature extraction in learning local traffic patterns. Excluding the BiLSTM layer yields an accuracy of 88.87%, suggesting that bidirectional temporal dependency modeling improves attack detection. The largest performance degradation is observed when the attention mechanism is removed, reducing the classification accuracy from 89.04% to 87.44%. This result highlights the significant contribution of the attention layer to the overall detection performance. The observed accuracy reduction indicates that not all temporal representations learned by the BiLSTM contribute equally to the final decision. By assigning higher weights to the most informative temporal features and suppressing less relevant representations, the attention mechanism enables the model to focus on critical traffic patterns associated with different attack categories. Consequently, it enhances class-level discrimination, reduces misclassification among similar attacks, and improves the overall effectiveness of the intrusion detection system.

Table 6 presents the computational complexity and performance analysis of the proposed IDS model. The results indicate that the proposed IDS is well-suited for deployment in consumer electronics environments. The training stage is computationally intensive, with a training time of 3301.1489 s and an additional ACO optimization time of 2129.67 s. However, these steps are intended to be executed offline on a resource-rich platform such as a server or an edge gateway. After offline training, the resulting model is compact, requiring only 1.2334 MB and 323,339 parameters, making it suitable for resource-constrained CE devices. The online detection stage is efficient, achieving an inference time of 0.0004269 s per sample and a mean batch inference time of 0.103346 s, enabling near real-time operation with minimal latency. In terms of detection performance, the model achieves a high macro one-vs-rest AUC of 0.9825, indicating strong class separability.

**Table 6:** Computational Complexity and Performance Analysis of the Proposed IDS Model.

Category	Metric	Value
Time	Training Time (s)	3301.1489
	ACO optimization time (s)	2129.67
	Total Inference time (s)	5.2728
	Inference time per sample (s/sample)	0.0004269
	Mean batch inference time (s/batch)	0.103346
	Std batch inference time (s/batch)	0.02614355
CPU/ Memory	Run time CPU usage (%)	57.4
	Peak Memory usage (MB)	518.5823
	Approx. model size (MB)	1.2334
	G-mean	0.0024849
	AUC OVR (macro)	0.9825
	PR-AUC (macro)	0.5865
	Total number of parameters	323339
	F1 macro	0.5294
	Recall macro	0.53566
	FLOPs	6031552
	Inference latency per sample (ms/sample)	0.2132

In contrast, the macro PR-AUC of 0.5865 and macro F1 and recall values of 0.5294 and 0.53566 indicate that the model exhibits lower detection performance for minority attack categories. This behavior can be attributed to the highly imbalanced nature of the UNSW-NB15 dataset, where several attack classes contain substantially fewer samples than the majority classes. Consequently, the classifier tends to achieve higher performance on dominant traffic categories while showing reduced sensitivity toward rare attacks. Despite this limitation, the proposed framework demonstrates effective overall classification capability and improved feature learning through ACO-based feature selection and attention-guided spatiotemporal modeling.

## Conclusion

This study proposes a hybrid intrusion detection framework to secure consumer electronics devices in smart CE networks. The proposed model integrates a DCNN with a BiLSTM to achieve accurate threat recognition and is evaluated on the UNSW-NB15 dataset. Experimental results demonstrate an accuracy of 89.04%, outperforming comparable techniques reported in the literature. To reduce computational complexity, an ACO-based feature subset selection strategy is employed, thereby decreasing input dimensionality and reducing inference overhead. Owing to its compact footprint, the proposed method is suitable for deployment on CE devices and CE edge gateways for real-time intrusion detection after offline training. However, model training and feature optimization remain computationally intensive and are therefore conducted offline on resource-rich systems.

Despite these advantages, the current study has three main limitations. First, the evaluation is restricted to a single dataset, which may limit the generalizability of the proposed framework across diverse network environments and traffic patterns. Second, the deployment analysis was conducted only in a software-based experimental environment. Consequently, hardware-specific performance metrics, such as inference latency, memory consumption during deployment, and energy efficiency on actual edge or consumer electronics devices, were not evaluated and remain subjects for future investigation. Third, the model shows reduced effectiveness in detecting minority attack classes, primarily due to class imbalance and limited samples for rare intrusions. Future work will focus on evaluating the proposed framework across additional IDS benchmark datasets and exploring more advanced deep learning architectures to further improve robustness, generalization capability, and overall detection performance. Furthermore, class-balancing techniques, cost-sensitive learning strategies, and data augmentation methods will be investigated to enhance the detection of minority attack classes while maintaining low computational complexity and efficient deployment characteristics.

## Supporting information

S1 File Antcolony code. (ZIP)
